# Direct Oral Administration of Cold-Pressed *Nigella sativa* Oil in Neonatal Lambs: A Longitudinal Assessment of Systemic Safety and Hematobiochemical Responses

**DOI:** 10.3390/vetsci13090862

**Published:** 2026-08-25

**Authors:** Ahmet Cihat Tunç, Emre Kaya, Sercan Hüseyin Bayendur, Fatih Mehmet Birdane

**Affiliations:** Department of Internal Medicine, Faculty of Veterinary Medicine, Afyon Kocatepe University, 03200 Afyonkarahisar, Turkey; kayaemre2013@gmail.com (E.K.); fbirdane@aku.edu.tr (F.M.B.)

**Keywords:** black cumin oil, hematology, lipid profile, neonatal lambs, *Nigella sativa*, systemic tolerance

## Abstract

Evaluating the in vivo systemic tolerance of phytogenic feed additives is a crucial prerequisite before their widespread application in vulnerable newborn ruminants. This study aimed to investigate the systemic effects of orally administered cold-pressed *Nigella sativa* oil on the hematological and biochemical profiles of neonatal lambs (0–45 days of age) fed exclusively on colostrum and maternal milk. The pre-specified primary endpoint was the absence of clinically meaningful change in hepatorenal markers and complete blood count between groups over 45 days. Thirty-two single-born Merino lambs were divided into a treatment group that received 1.5 mL/day of the oil for the first 15 days of life and an untreated control group. Blood samples were collected on days 0, 7, 21, and 45 to evaluate both immediate and carry-over physiological effects. Using a covariate-adjusted linear mixed-effects model to account for baseline variances, the results demonstrated that the oil was physiologically well-tolerated. The supplementation exerted no significant adverse effects (*p* > 0.05) on hepatorenal markers or the measured acute-phase protein. The supplementation maintained neonatal energy metabolism stability without inducing artificial lipid suppression in serum triglycerides or total cholesterol levels. Furthermore, the oil preserved hematological homeostasis without negatively impacting red blood cell indices and modulated platelet distribution width indices without causing pathological thrombocytosis. In conclusion, the early direct oral administration of *N. sativa* oil is a physiologically well-tolerated supplement that supports basal metabolic and hematological stability in pre-ruminant lambs without inducing systemic disruptions within the parameters measured.

## Simple Summary

Newborn lambs possess underdeveloped digestive and immune systems, making them highly vulnerable to environmental and physiological stressors. To support their early health, natural plant-based supplements like cold-pressed black cumin (*Nigella sativa*) oil are of great interest due to their potential antioxidant properties. However, because these liquid supplements pass directly into the abomasum of pre-ruminant newborns without being broken down in the rumen, establishing their fundamental systemic safety during the critical early developmental phase (0–45 days) is a crucial first step. This study aimed to evaluate the physiological tolerance of directly administered black cumin oil in healthy newborn lambs. We administered the oil for the first 15 days of life to provide early support, and monitored their blood profiles and overall health up to 45 days of age to assess both immediate and carry-over effects compared to an untreated group. The results demonstrated that no adverse effects were detected within the parameters measured in this study. The supplementation safely maintained hematological homeostasis and hepatorenal markers without negatively altering blood lipids. In conclusion, the early administration of black cumin oil is physiologically well-tolerated in pre-ruminant lambs, supporting their basal metabolism without inducing systemic disruptions.

## 1. Introduction

Newborn ruminants undergo rapid anatomical and functional development during their first weeks of life, a period marked by heightened vulnerability to environmental and physiological stressors [1,2]. Even under optimal herd management conditions—characterized by healthy ewes, well-managed gestations, and adequate colostral intake—neonates face a physiological “immunity gap” as maternally derived antibody levels decline while their endogenous immune system is still maturing. Within this specific window, their immature hepatorenal and immune systems exhibit high susceptibility to oxidative stress [3]. Consequently, supplementing the basal milk diet with natural antioxidant and immunomodulatory compounds during these critical early weeks is essential to bridge this physiological gap and promote early cellular homeostasis [4].

Phytogenic feed additives, including cold-pressed *Nigella sativa* (black cumin) oil, have recently garnered substantial interest owing to their potent antioxidant and anti-inflammatory properties, particularly given their unique absorptive pathways in pre-ruminants [5,6,7]. The oil itself is highly valued for its active phytoconstituents, notably thymoquinone [8,9,10,11]. As a lipid source, it is rich in polyunsaturated fatty acids (PUFAs), predominantly linoleic acid, which elevates the omega-6 fraction and results in an imbalanced omega-6/omega-3 ratio [12]. Nevertheless, its high concentration of bioactive compounds exerts a robust protective effect against systemic inflammation by suppressing oxidative stress at the cellular level [13,14,15,16,17].

Although the beneficial effects of *N. sativa* are well documented in adult ruminants—where it acts primarily as a rumen modifier to enhance fermentation and growth performance—its application in neonatal pre-ruminants represents a fundamentally distinct physiological paradigm [18,19,20,21,22]. In neonates, the reticular (esophageal) groove reflex shunts ingested liquids directly into the abomasum, completely bypassing the reticulorumen [7]. Consequently, orally administered phytogenic oils escape ruminal microbial biohydrogenation. This absence of ruminal degradation allows unsaturated fatty acids and sensitive phytoconstituents to reach the lower gastrointestinal tract intact, thereby profoundly altering their systemic absorption and bioavailability compared with adult ruminants.

Because these highly bioavailable bioactive constituents bypass the rumen and enter systemic circulation directly, establishing their baseline in vivo systemic safety and physiological tolerance within the unique pre-ruminant anatomy constitutes a critical prerequisite prior to their widespread prophylactic adoption [23]. We therefore hypothesized that, owing to the absence of ruminal biohydrogenation, direct oral administration of cold-pressed *N. sativa* oil would result in systemic absorption while remaining physiologically well-tolerated, thereby supporting hematobiochemical stability without inducing hepatorenal toxicity in pre-ruminant neonates. Accordingly, rather than conducting a performance or therapeutic efficacy trial, the objective of this study was to comprehensively evaluate the systemic effects and physiological tolerance of directly administered *N. sativa* oil on the hematological and biochemical profiles of healthy neonatal lambs prior to full functional maturation of the rumen.

## 2. Materials and Methods

### 2.1. Study Design and Ethical Approval

This prospective, controlled, longitudinal study was conducted in strict accordance with the ARRIVE (Animal Research: Reporting of In Vivo Experiments) guidelines. The experimental protocol and animal welfare risk assessments were formally reviewed and approved by the Ethics Committee on Animal Experiments of Afyon Kocatepe University (Approval No. 49533702/43). The field trial was conducted at a commercial livestock enterprise operating under intensive production conditions in Afyonkarahisar, Türkiye.

### 2.2. Study Population, Housing, and Selection Criteria

A total of 32 newborn Merino lambs were enrolled in the study. To minimize confounding maternal and physiological variables, stringent inclusion and exclusion criteria were applied:

1. All lambs were born to healthy, multiparous Merino ewes of uniform age (2–3 years old).
2. Only single-born (monotocous) lambs were included. Ewes with twin or multiple pregnancies were strictly excluded to eliminate confounding variations resulting from birth weight disparities and uneven maternal milk allocation.
3. The animals were assigned to two experimental cohorts: the *N. sativa* oil-treated group (*n* = 19) and the untreated healthy control group (*n* = 13). The numerical imbalance between groups reflected the flock’s natural single-birth distribution during that lambing season. In compliance with ARRIVE guidelines, simple randomization was performed immediately following the birth of each eligible single-born lamb. Group allocation was executed sequentially via unstratified drawing of lots from a blinded container. Despite the unstratified design, post hoc evaluation confirmed no baseline imbalances between the final groups regarding sex distribution, dam characteristics, or initial colostrum intake. This simple unstratified allocation accounts for the unequal, yet entirely random, group sample sizes in this exploratory pilot trial.

Throughout the 15-day experimental period, lambs were housed in group pens alongside their dams in a naturally ventilated facility on deep wheat-straw bedding. Both ewes and lambs had ad libitum access to fresh water. Ewes were fed a standard basal total mixed ration (TMR) ad libitum, formulated to meet or exceed core nutrient requirements, without growth promoters or supplementary feed additives. The maternal basal diet comprised corn silage, wheat straw, alfalfa hay, barley meal, corn meal, cottonseed meal, soybean meal, wheat bran, third-grade wheat flour (razmol), and a commercial vitamin–mineral premix. The approximate chemical and nutritional composition of this maternal ration was: crude protein, 16.0%; crude fat, 4.2%; crude fiber, 10.5%; and metabolizable energy, 11.5 MJ/kg on a dry matter (DM) basis. Newborn lambs were nourished exclusively on colostrum and maternal milk, alongside ad libitum water intake, throughout the study.

### 2.3. Protocols and Dietary Intervention

Lambs assigned to the treatment group (*n* = 19) received a daily oral dose of 1.5 mL of cold-pressed *Nigella sativa* (black cumin) oil for 15 consecutive days, starting immediately following the initial colostrum-feeding phase. The fatty acid profile of the oil, determined using gas chromatography–flame ionization detection (GC-FID), comprised 14.6% hexadecanoic acid, 3.1% octadecanoic acid, 22.3% (*Z*)-9-octadecenoic acid, 55.0% (*Z*,*Z*)-9,12-octadecadienoic acid, and 2.6% (*Z*,*Z*)-11,14-eicosadienoic acid, alongside 2.4% minor unlisted fatty acids. Total fatty acid proportions were determined to be 57.6% total omega-6, 22.3% total omega-9, 79.9% total unsaturated fatty acids, and 17.7% total saturated fatty acids (Table 1).

The daily dosage of 1.5 mL was established via allometric scaling based on body surface area (BSA = BW^0.67^) derived from a verified reference dose (5.0 mL/day) documented to be clinically safe and immunomodulatory in newborn buffalo calves [24]. Based on an estimated mean birth weight of 30 kg for newborn buffalo calves (BSA ≈ 9.83 kg^0.67^), the reference surface-area dosage equates to 0.508 mL/kg^0.67^. Extrapolating this factor to the Merino lambs in the treatment group, which had a recorded mean baseline birth weight of 4.78 kg (BSA ≈ 2.85 kg^0.67^) (Table 1), the target dose was calculated as follows: (0.508 mL/kg^0.67^ × 2.85 kg^0.67^) ≈ 1.45 mL/day. This extrapolated value was rounded to a practical daily volume of 1.5 mL to facilitate precise dosing via oral syringe. To eliminate aspiration risk and strategically stimulate the esophageal groove reflex—thereby ensuring the fluid bypassed ruminal microbial biohydrogenation and passed directly into the abomasum—the oil was administered slowly using a graduated oral syringe placed at the caudal aspect of the tongue. Animals in the control group (*n* = 13) underwent a daily sham handling procedure to account for handling stress and administrative protocols. Daily clinical monitoring revealed no signs of gastrointestinal intolerance, oil-induced diarrhea, or systemic adverse reactions in any animal throughout the trial. Lambs in both groups were weighed immediately prior to each scheduled blood sampling to track individual growth trajectories.

### 2.4. Blood Sampling and Laboratory Analysis

Prior to blood sampling on each designated testing day, all lambs underwent a thorough clinical examination, including evaluation of rectal temperature, heart rate, and respiratory rate, to confirm overall health status. To evaluate both immediate and residual (carryover) physiological effects, serial fasting venous blood samples (collected prior to morning feeding) were obtained via jugular venipuncture from both cohorts at four distinct time points: Day 0 (baseline/pre-treatment), Day 7, Day 21, and Day 45. Blood collection on Day 15 (the final day of treatment) was intentionally omitted to avoid acute handling stress, thereby allowing a clearer assessment of short-term carryover tolerance on Day 21. For hematological profiling, blood was collected into vacuum tubes containing EDTA and analyzed immediately using a HumaCount^®^ 80TS automated hematology analyzer (Human Diagnostics, Wiesbaden, Germany). For serum biochemical analysis, parallel blood samples were drawn into vacuum tubes containing a clot activator and gel separator. The samples were allowed to clot at room temperature and subsequently centrifuged at 5000 rpm for 5 min to yield serum, which was stored at −20 °C until analysis. Serum concentrations of glucose, total protein, albumin, cholesterol, urea, total bilirubin, triglycerides, low-density lipoprotein (LDL), high-density lipoprotein (HDL), alanine aminotransferase (ALT), aspartate aminotransferase (AST), alkaline phosphatase (ALP), gamma-glutamyl transferase (GGT), and C-reactive protein (CRP) were quantified spectrophotometrically using a Randox^®^ Monaco automated biochemistry analyzer (Randox Laboratories, Crumlin, UK). Routine internal quality control and daily calibration procedures for both automated analyzers were strictly performed using standard commercial control sera prior to sample evaluation. As an exploratory in vivo pilot study, routine hematological indices and CRP were prioritized as primary screening parameters owing to logistical and budgetary constraints; specific ovine acute-phase proteins (e.g., haptoglobin and serum amyloid A [SAA]) were not evaluated in this baseline panel.

### 2.5. Statistical Analysis

Statistical analyses were performed using the Python programming language (version 3.10). The assumption of normality for continuous variables was verified by evaluating model residuals via the Shapiro–Wilk test, and homogeneity of variances across groups was assessed using Levene’s test. Descriptive statistics are expressed as mean ± standard deviation (SD). To account for intra-individual correlations inherent to the longitudinal, repeated-measures design, data were analyzed using a linear mixed-effects model (LMM) framework, fitted via the Restricted Maximum Likelihood (REML) approach. Implementing the LMM framework was essential as it preserves statistical power and variance stability in unbalanced experimental designs (*n* = 19 versus *n* = 13), thereby preventing the inflation of Type I errors typical of traditional repeated-measures ANOVA under unequal sample sizes. To properly adjust for pre-treatment variations and align with the physiological nature of the timeline, baseline blood samples (Day 0) were incorporated into the model as a continuous covariate. Consequently, the dietary intervention (Group: Control vs. Black Cumin Oil), sampling time points (Time: Days 7, 21, and 45), and their interaction (Group × Time) were defined as fixed effects. The individual lamb identification numbers were specified as a random effect to model the subject-specific covariance structure over time. For parameters exhibiting statistically significant Group × Time interactions, post hoc pairwise comparisons were performed on the Estimated Marginal Means (EMMs) utilizing a Bonferroni adjustment to identify the specific time points driving the interaction. Furthermore, to address the magnitude of the intervention and ensure adequate post hoc model power, effect sizes were reported using Nakagawa’s R^2^; yielding both Marginal R^2^ ($R_{m}^{2}$; variance explained by fixed factors) and Conditional R^2^ ($R_{c}^{2}$; total variance explained by both fixed and random factors). For all statistical tests, the significance threshold was set at *p* < 0.05.

### 2.6. The Use of Artificial Intelligence for Linguistic Refinement

During the preparation of this manuscript, the software tool Grammarly was utilized strictly for grammar correction, linguistic refinement, and improving the overall readability of the text. No automated tools were used in data analysis, result interpretation, or the drawing of scientific conclusions. The authors thoroughly reviewed, edited, and approved all texts and take full and absolute responsibility for the publication’s final content.

## 3. Results

### 3.1. Baseline Values and Growth Performance

In the linear mixed-effects model analysis, where Day 0 values were incorporated as a baseline covariate to adjust for initial variance, the effect of the baseline covariate was highly significant (P_Cov_ < 0.001). Over the 45-day monitoring period, the body weights of lambs in both groups increased in accordance with expected physiological growth trajectories, exhibiting a highly significant main effect of time (P_Time_ < 0.001). However, no significant main effect of black cumin oil administration (P_Group_ = 0.669) or interaction effect (P_Group×Time_ = 0.705) was observed for body weight gain (Table 2).

### 3.2. Biochemical Profile, Hepatorenal, and Acute-Phase Protein (CRP) Markers

The effects of *N. sativa* oil administration on hepatorenal function and systemic inflammation markers are summarized in Table 3. Serum C-reactive protein (CRP) levels, total bilirubin concentrations, and liver enzymes reflective of hepatocellular and biliary integrity (ALT, AST, and GGT) did not differ significantly between groups (P_Group_ > 0.05). There was no significant between-group difference in CRP at any time point, although CRP increased in both groups over time, consistent with the known age-related rise in baseline CRP in neonatal lambs. Similarly, pancreatic lipase activity showed no significant treatment effect (P_Group_ = 0.393). While time-dependent shifts were statistically significant across all evaluated parameters throughout the 45-day monitoring period (P_Time_ < 0.05), no significant Group × Time interaction was observed. For blood urea nitrogen (BUN), a marker of renal function, a significant main effect of group was observed (P_Group_ < 0.021), whereas the group × time interaction was not significant (P_Group×Time_ = 0.533). Regarding alkaline phosphatase (ALP) activity, significant main effects were observed for both group (P_Group_ = 0.025) and time (P_Time_ < 0.001); however, baseline covariate adjustment confirmed that the group × time interaction was not significant (P_Group×Time_ = 0.099), indicating parallel temporal trajectories between the cohorts.

### 3.3. Lipid Profile

Evaluation of the serum lipid profile (Table 3) revealed no significant differences between *N. sativa* oil-treated lambs and the control group regarding total cholesterol (P_Group_ = 0.843), HDL (P_Group_ = 0.832), or LDL (P_Group_ = 0.263) concentrations; only time-dependent variations associated with physiological development were significant (P_Time_ < 0.001). Similarly, analysis of triglyceride metabolism demonstrated a highly significant main effect of time (P_Time_ < 0.001), reflecting normal physiological maturation, whereas the main effect of group (P_Group_ = 0.568) and the Group × Time interaction (P _Group×Time_ = 0.801) were non-significant.

### 3.4. Hematological Parameters

The hematological profile of the lambs is presented in Table 4. No significant treatment effect was observed on absolute counts of total white blood cells, lymphocytes, monocytes, or granulocytes (P_Group_ > 0.05). Similarly, erythrocyte parameters—including RBC, hemoglobin, hematocrit, MCV, MCH, MCHC, RDVs, and RDWc—showed no significant differences between cohorts (P_Group_ > 0.05). Likewise, primary platelet parameters, including PLT (P_Group_ = 0.894), PCT (P_Group_ = 0.873), and MPV (P_Group_ = 0.703), did not differ between *N. sativa* oil-treated lambs and control animals.

However, significant group × time interactions were identified for the platelet distribution width indices, PDWs (P_Group×Time_ = 0.010) and PDWc (P_Group×Time_ = 0.026). Bonferroni-adjusted post hoc pairwise comparisons of estimated marginal means revealed no isolated between-group differences at specific sampling time points (Days 7, 21, or 45; all P_adj_ > 0.05). This indicates that these interactions were driven by divergent temporal trajectories between the cohorts rather than absolute cross-sectional disparities at individual sampling points. Furthermore, a significant main effect of time (P_Time_ < 0.05) was detected across all primary hematological indices—excluding differential mid-cell percentages and MPV—reflecting normal physiological development.

## 4. Discussion

Dietary supplementation with *Nigella sativa* (as seeds, meal, or oil) often acts as a natural growth promoter, improving average daily gain and feed efficiency during the active fattening period [25,26,27,28,29]. However, our study observed no significant differences in body weight gain. The lack of a statistically significant difference in body weight gain following the direct administration of black cumin oil in our study should be evaluated in light of the variable findings in the literature, particularly in the context of neonatal (pre-ruminant) physiology. Nearly all of the aforementioned studies reporting distinct positive effects were conducted on weaned ruminants with fully developed rumen flora during their active fattening period (2 to 10 months of age). This lack of a growth-promoting effect can be directly explained by the pre-ruminant physiology of the neonates. During the 0–45-day period, the rumen is non-functional. The administered oil bypassed microbial biohydrogenation via the esophageal groove reflex, reaching the abomasum directly. Without a developed rumen microbiota to modulate, the expected anabolic mechanisms were absent. The parallel growth trajectories simply reflect the neonates’ basal nutritional intake from maternal milk, demonstrating that the direct abomasal absorption of the oil did not induce osmotic malabsorption or disrupt gastrointestinal energy utilization.

The systemic absorption of concentrated phytogenic supplements carries the potential to create a toxic metabolic load on the immature neonatal liver and kidneys. However, our biochemical results demonstrated physiological tolerance. Regarding hepatic and biliary function, the direct administration of *N. sativa* oil did not alter the activities of ALT, AST, or GGT. The enzyme dynamics of ALP, a marker of bone formation and biliary metabolism, also support the physiological adaptation toward growth and cellular construction. The absence of any statistical difference between the groups in total bilirubin levels and the enzyme activities of GGT, ALT, and AST—the most specific indicators of hepatocellular and biliary injury—conclusively excludes the possibility that the changes detected in the ALP enzyme originated from the liver or biliary tract. In light of this clinical elimination, the strong main effect of time across all groups indicates that the offspring’s skeletal and intestinal systems developed rapidly over the 45 days. Regarding ALP activity, although the main effect of group and the main effect of time were statistically significant, the covariate adjustment revealed that the Group × Time interaction was not significant, indicating parallel temporal trajectories between the groups. While these parallel dynamics might be hypothesized to relate to osteoblastic mineralization during neonatal growth, it is crucial to note that in neonatal ruminants, ALP also originates from intestinal isoforms in addition to bone [30]. Therefore, the hypothesis that *N. sativa* oil actively participates in growth metabolism via bone cannot be conclusively supported without bone-specific ALP isoform quantification or complementary bone turnover markers. These mechanisms remain an important hypothesis to be robustly tested in future studies. Because these enzymes are specific intracellular markers of hepatocellular injury, their strict stability confirms that the neonatal liver successfully metabolized the lipid load without undergoing cellular degeneration. The biochemical basis of this observed hepatorenal tolerance might lie in *N. sativa*’s potent antioxidant capacity [27], as well as the free radical-scavenging and tissue-protective properties of its active phytochemicals such as thymoquinone, carvacrol, anethole, and 4-terpineol [31,32]. This protective mechanism perfectly aligns with previous studies where black cumin supplementation safely maintained transaminase enzymes and overall hepatic functions in newborn buffalo calves [24] and Farafra lambs [28]. Since BUN is an important indicator of amino acid metabolism in ruminants [27,33], although a covariate-adjusted statistical main effect of group was observed for BUN, the Group × Time interaction was strictly non-significant, and all measured values remained consistently within healthy physiological reference intervals. Therefore, this isolated effect does not indicate any clinical renal impairment or toxic metabolic load. This confirmed renal safety is consistent with the lack of prominent adverse alterations in blood parameters reported in ewes supplemented with black cumin [34].

In addition to toxicological safety, the effects of black cumin supplementation on systemic inflammation were also evaluated. No increase in CRP levels was observed with black cumin administration, which, despite being a minor acute-phase protein in sheep, provides supportive data regarding the inflammatory course in clinical biochemistry panels. The stable course of CRP, when combined with the absence of pathological fluctuations in leukocyte counts and granulocyte-to-lymphocyte ratios in the hemogram, can be considered an indicator that the administered oil does not induce reactive inflammation or immunological stress at the cellular level. However, this conclusion is constrained by a notable methodological limitation. Although the stable course of CRP and the lack of pathological deviations in the leukocyte differential strongly support systemic tolerance, haptoglobin and serum amyloid A (SAA)—which are the primary major acute-phase reactants in sheep—were not measured. We explicitly acknowledge that the absence of these specific major markers constrains our definitive conclusions regarding absolute inflammatory safety. The inclusion of these major acute-phase proteins in future in vivo panels will elucidate the immunological tolerance profile of the oil during the neonatal period in much greater detail.

When evaluating the lipid profile dynamics, the covariate-adjusted main effect of the administration and the Group × Time interaction were found to be strictly non-significant for triglyceride, total cholesterol, HDL, and LDL levels. Instead, a highly significant main effect of time was revealed, corresponding to the natural physiological maturation of the neonates. This stability contrasts with the highly variable results reported in older ruminants, where *N. sativa* often exerts hypolipidemic effects by suppressing cholesterol production and accelerating lipid metabolism [28,35,36,37,38], though some studies similarly report no reduction or even increases in lipid levels [20,21,26,37,39]. We interpret our lack of lipid suppression as a physiological prioritization of the neonatal anabolic drive. Unlike adult mammals, neonates in a rapid growth phase require high amounts of structural cholesterol and lipoproteins to synthesize millions of new cell membranes and complete central nervous system myelination, a critical requirement also reported in human preterm newborns [40,41]. Our findings demonstrate that directly orally administered *N. sativa* oil does not induce an artificial lipid suppression or exert a persistent pharmacological carry-over effect, but safely preserves the vital basal energy cycle and structural cholesterol requirement of the offspring during the critical 45-day pre-ruminant period.

In the hematological profile, the direct administration of *N. sativa* oil maintained systemic stability without negatively impacting red blood cell indices. Specifically, the lack of significant differences in Hb and HCT values indicates that the oil safely supported the high anabolic growth rate and increasing tissue oxygenation demand of the neonates. This observed hematological stability is consistent with previous reports in sheep [42], contrasting with the HCT elevations occasionally observed in supplemented calves [29]. Furthermore, the absence of any pathological deviation in WBCs and their subgroups perfectly aligns with our CRP findings. This harmony confirms the lack of a reactive systemic infection or inflammatory state at the cellular level, corroborating the safe leukocyte profiles previously reported in growing lambs fed black cumin meal [29]. The most distinct hematological finding was the highly statistically significant Group × Time interaction observed in platelet distribution width (PDW) indices. Since platelet indices are sensitive biomarkers reflecting megakaryocytic activity and bone marrow dynamics, this temporal shaping of PDW must be interpreted alongside the stable CRP and leukocyte profiles. In this non-inflammatory environment, the PDW interaction indicates that the oil does not induce a systemic reactive thrombocytosis. Instead, it suggests a controlled immuno-hematopoietic modulation, likely driven by thymoquinone’s known ability to stimulate macrophage activity and T-helper cells [43,44]. Crucially, this targeted cellular stimulation occurred without a pathological increase in total PLT, maintaining platelet counts within healthy physiological limits. Consequently, these findings suggest that alongside its basal nutritional contribution, the direct oral administration of *N. sativa* oil safely modulates platelet dynamics over time without inducing reactive thrombocytosis or systemic inflammatory stress.

The highly significant main effect of time observed across the vast majority of parameters in our study is not a confounding factor or a side effect of the administered *N. sativa* oil. Rather, it is a natural reflection of the rapid cellular maturation and anabolic growth momentum of the lambs during the 0–45-day pre-ruminant period. Because the rumen is not yet functional during this early phase, and liquid *N. sativa* oil bypasses microbial biohydrogenation via the esophageal groove reflex to reach the abomasum directly, evaluating systemic biochemical and hematological tolerance—instead of rumen fermentation parameters—was the fundamental rational strategy of this study. The fact that the oil administration operated in parallel with this intense time-dependent physiological maturation without causing disruption confirms its systemic safety.

## 5. Conclusions

In conclusion, this study establishes the systemic tolerance of directly orally administered cold-pressed *Nigella sativa* oil at a dose of 1.5 mL/day for 15 consecutive days in healthy neonatal Merino lambs. Based strictly on the hematological and biochemical parameters evaluated over the 45-day pre-ruminant observation period, the supplementation did not act as a direct growth promoter, but it successfully preserved the expected physiological growth trajectories of the neonates. The administered dose exerted no adverse effects on hepatorenal integrity, as evidenced by stable ALT, AST, GGT, and BUN levels. Furthermore, the oil safely maintained basal lipid homeostasis without inducing artificial lipid suppression and supported hematological stability—including erythrocyte, leukocyte, and platelet indices—without inducing systemic inflammatory stress within the scope of the measured CRP levels. Therefore, our findings specifically demonstrate that *N. sativa* oil is a clinically safe and well-tolerated dietary intervention for neonatal lambs under these experimental conditions. Establishing this foundational safety profile provides a crucial stepping stone for future long-term studies incorporating major acute-phase proteins and comprehensive coagulation panels to further explore its functional applications in ruminant health.

## Figures and Tables

**Table 1:** Fatty acid profile of the cold-pressed *Nigella sativa* oil.

Fatty Acid	Percentage (%)
Hexadecanoic acid	14.6
Octadecanoic acid	3.1
(*Z*)-9-octadecenoic acid	22.3
(*Z*,*Z*)-9,12-octadecadienoic acid	55.0
(*Z*,*Z*)-11,14-eicosadienoic acid	2.6
Minor unidentified fatty acids	2.4
Total	100.0

**Table 2:** Body weight values between the black cumin oil and the control groups.

Day		0 (Cov)	7	21	45	P_Time_	P_Group_	P_Group×Time_	$R_{m}^{2}$	$R_{c}^{2}$
Weighing (kg)	Black Cumin Oil	4.78 ± 0.95	7.13 ± 1.54	9.28 ± 2.54	13.41 ± 3.88	<0.001	0.669	0.705	0.730	0.849
	Control	4.55 ± 0.90	6.90 ± 1.35	9.25 ± 1.93	12.76 ± 4.00					

0, 7, 21, and 45 represent the weighing days.

**Table 3:** Biochemical profiles of neonatal lambs receiving cold-pressed black cumin oil (*n* = 19) versus control lambs (*n* = 13).

Day		0 (Cov)	7	21	45	P_Time_	P_Group_	P_Group×Time_	$R_{m}^{2}$	$R_{c}^{2}$
CRP (mg/L)	Black cumin Oil	4.15 ± 0.74	4.23 ± 0.60	4.77 ± 0.61	5.22 ± 0.82	<0.001	0.439	0.123	0.432	0.651
	Control	3.96 ± 0.79	3.99 ± 1.04	4.76 ± 0.80	5.55 ± 0.60					
BUN (mg/dL)	Black cumin Oil	21.78 ± 9.54	18.12 ± 9.38	21.97 ± 8.40	14.85 ± 2.22	<0.001	0.021	0.533	0.239	0.328
	Control	18.26 ± 10.06	12.76 ± 3.91	17.82 ± 3.41	12.79 ± 4.29					
ALT (U/L)	Black cumin Oil	9.90 ± 2.02	8.68 ± 2.01	6.14 ± 3.18	5.03 ± 3.48	<0.001	0.262	0.876	0.215	0.215
	Control	8.47 ± 3.35	7.10 ± 4.17	5.60 ± 2.67	4.49 ± 1.93					
AST (U/L)	Black cumin Oil	74.52 ± 17.43	50.12 ± 8.65	37.66 ± 9.84	58.84 ± 20.00	<0.001	0.562	0.501	0.284	0.318
	Control	63.23 ± 20.80	51.73 ± 10.01	47.12 ± 21.79	62.03 ± 12.84					
ALP (U/L)	Black cumin Oil	909.74 ± 553.22	664.37 ± 290.76	501.47 ± 204.25	313.32 ± 176.95	<0.001	0.025	0.099	0.428	0.560
	Control	658.46 ± 240.88	825.23 ± 152.28	463.08 ± 189.68	346.92 ± 219.53					
GGT (U/L)	Black cumin Oil	649.07 ± 453.13	172.53 ± 77.23	81.76 ± 21.92	82.67 ± 20.21	<0.001	0.310	0.453	0.486	0.607
	Control	594.96 ± 226.09	172.72 ± 90.30	88.01 ± 29.99	84.41 ± 26.86					
TRIG (mg/dL)	Black cumin Oil	102.47 ± 27.23	84.42 ± 30.21	47.37 ± 22.72	54.63 ± 21.70	<0.001	0.568	0.801	0.291	0.294
	Control	87.92 ± 29.61	87.85 ± 26.15	48.00 ± 27.65	64.00 ± 26.82					
CHOL (mg/dL)	Black cumin Oil	82.86 ± 21.26	82.06 ± 18.58	88.00 ± 23.71	45.31 ± 14.63	<0.001	0.843	0.199	0.505	0.685
	Control	78.23 ± 18.29	79.76 ± 16.68	81.49 ± 19.71	53.05 ± 17.31					
HDL (mg/dL)	Black cumin Oil	28.67 ± 9.82	39.64 ± 13.49	43.50 ± 17.79	21.43 ± 9.71	<0.001	0.832	0.437	0.332	0.498
	Control	28.60 ± 9.01	39.62 ± 7.97	40.96 ± 16.99	27.11 ± 15.74					
LDL (mg/dL)	Black cumin Oil	30.33 ± 13.48	26.47 ± 10.21	32.82 ± 10.43	18.13 ± 10.08	<0.001	0.263	0.595	0.369	0.479
	Control	30.78 ± 13.91	21.95 ± 12.56	33.33 ± 13.33	16.93 ± 9.06					
Lipase (mg/dL)	Black cumin Oil	36.63 ± 13.80	49.05 ± 29.26	26.84 ± 20.73	35.63 ± 21.61	0.004	0.398	0.736	0.221	0.221
	Control	30.85 ± 11.17	40.23 ± 21.89	20.00 ± 14.01	21.38 ± 7.92					

ALP: Alkaline Phosphatase; ALT: Alanine Aminotransferase; AST: Aspartate Aminotransferase; GGT: Gamma-Glutamyl Transferase; TRIG: Triglycerides; CHOL: Cholesterol; HDL: High-Density Lipoprotein; LDL: Low-Density Lipoprotein; BUN: Blood Urea Nitrogen; CRP: C-Reactive Protein; 0, 7, 21, and 45 represent the blood sampling days.

**Table 4:** Hematological parameters of neonatal lambs receiving cold-pressed black cumin oil (*n* = 19) versus control lambs (*n* = 13).

Day		0 (Cov)	7	21	45	P_Time_	P_Group_	P_Group×Time_	$R_{m}^{2}$	$R_{c}^{2}$
RBC (10^12^/L)	Black cumin Oil	8.56 ± 1.88	7.87 ± 1.20	6.91 ± 1.89	9.68 ± 2.28	<0.001	0.313	0.797	0.324	0.353
	Control	9.08 ± 1.45	8.61 ± 1.20	8.32 ± 2.49	10.76 ± 1.70					
WBC (10^9^/L)	Black cumin Oil	5.22 ± 1.51	5.34 ± 2.70	7.84 ± 2.66	9.01 ± 4.07	<0.001	0.338	0.554	0.293	0.309
	Control	4.69 ± 1.47	6.07 ± 2.74	6.86 ± 2.84	8.96 ± 3.26					
Lymp (10^9^/L)	Black cumin Oil	3.12 ± 0.84	3.25 ± 1.54	5.02 ± 1.80	5.66 ± 1.59	<0.001	0.535	0.414	0.326	0.371
	Control	2.70 ± 0.65	3.46 ± 1.33	4.84 ± 1.91	6.54 ± 2.15					
MID (10^9^/L)	Black cumin Oil	0.36 ± 0.18	0.56 ± 0.69	0.68 ± 0.36	0.74 ± 0.47	0.222	0.652	0.807	0.240	0.534
	Control	0.36 ± 0.18	0.48 ± 0.18	0.49 ± 0.25	0.61 ± 0.38					
Gran (10^9^/L)	Black cumin Oil	1.72 ± 1.01	1.52 ± 0.76	2.10 ± 1.45	2.61 ± 1.68	0.245	0.484	0.338	0.143	0.297
	Control	1.63 ± 1.00	2.11 ± 1.59	1.53 ± 0.99	1.80 ± 1.28					
Hb (g/dL)	Black cumin Oil	11.32 ± 2.14	10.31 ± 1.09	9.69 ± 0.97	11.30 ± 1.77	<0.001	0.638	0.619	0.422	0.513
	Control	12.88 ± 1.64	10.95 ± 1.44	10.19 ± 0.64	11.51 ± 1.04					
MCH (pg)	Black cumin Oil	14.01 ± 0.88	12.60 ± 0.70	10.50 ± 0.71	10.03 ± 0.84	<0.001	0.654	0.627	0.721	0.820
	Control	13.75 ± 0.72	12.44 ± 0.68	10.50 ± 0.93	9.73 ± 0.59					
MCHC (g/dL)	Black cumin Oil	39.90 ± 2.15	37.78 ± 1.61	35.51 ± 1.78	36.70 ± 2.55	<0.001	0.796	0.888	0.302	0.535
	Control	39.70 ± 1.45	37.99 ± 1.09	36.01 ± 1.13	37.25 ± 1.97					
MCV (fL)	Black cumin Oil	35.18 ± 2.03	33.38 ± 2.19	29.63 ± 2.57	27.50 ± 3.50	<0.001	0.603	0.662	0.539	0.733
	Control	34.60 ± 1.78	32.82 ± 1.90	29.14 ± 2.76	26.20 ± 2.41					
RDWs (fL)	Black cumin Oil	15.26 ± 2.94	17.48 ± 3.68	14.66 ± 4.08	11.80 ± 3.51	<0.001	0.832	0.126	0.414	0.526
	Control	14.99 ± 1.99	17.75 ± 4.99	11.58 ± 3.64	9.05 ± 3.77					
RDWc (%)	Black cumin Oil	23.62 ± 1.68	26.91 ± 3.82	28.62 ± 3.94	29.35 ± 4.59	0.019	0.659	0.188	0.303	0.667
	Control	23.93 ± 0.98	27.50 ± 4.18	26.81 ± 3.41	28.77 ± 3.10					
HCT (%)	Black cumin Oil	28.45 ± 5.54	27.34 ± 3.41	27.33 ± 2.76	30.80 ± 4.46	<0.001	0.626	0.597	0.338	0.504
	Control	32.60 ± 5.21	28.85 ± 3.53	28.32 ± 1.80	31.04 ± 4.07					
PLT (10^9^/L)	Black cumin Oil	417.16 ± 113.35	613.32 ± 147.60	576.58 ± 146.83	615.00 ± 248.38	0.751	0.894	0.576	0.077	0.147
	Control	339.31 ± 82.20	609.38 ± 183.31	484.31 ± 182.32	539.46 ± 148.61					
PCT (%)	Black cumin Oil	0.23 ± 0.06	0.32 ± 0.08	0.29 ± 0.08	0.33 ± 0.14	0.478	0.873	0.623	0.088	0.174
	Control	0.19 ± 0.04	0.32 ± 0.09	0.25 ± 0.10	0.28 ± 0.08					
PDWs (fL)	Black cumin Oil	5.07 ± 0.75	4.55 ± 0.63	4.66 ± 0.75	5.42 ± 0.94	<0.001	0.855	0.010	0.288	0.426
	Control	5.86 ± 1.34	4.84 ± 0.66	5.44 ± 0.84	5.15 ± 0.88					
PDWc (%)	Black cumin Oil	31.75 ± 1.81	30.26 ± 1.89	30.67 ± 2.23	32.61 ± 2.44	<0.001	0.723	0.026	0.256	0.387
	Control	33.54 ± 2.65	31.16 ± 1.75	32.65 ± 2.35	31.91 ± 2.43					
MPV (fL)	Black cumin Oil	5.61 ± 0.43	5.21 ± 0.35	5.10 ± 0.37	5.35 ± 0.40	0.980	0.703	0.185	0.282	0.284
	Control	5.32 ± 1.39	5.26 ± 0.33	5.24 ± 0.26	8.99 ± 3.52					

WBC: White Blood Cell count; Lymp: Lymphocyte count; MID: Mid-size cell count; Gran: Granulocyte count; Hb: Hemoglobin; MCH: Mean Corpuscular Hemoglobin; MCHC: Mean Corpuscular Hemoglobin Concentration; RBC: Red Blood Cell count; MCV: Mean Corpuscular Volume; RDWs: Red Cell Distribution Width—standard deviation; RDWc: Red Cell Distribution Width—coefficient of variation; HCT: Hematocrit; PLT: Platelet count; PCT: Plateletcrit; PDWs: Platelet Distribution Width—standard deviation; PDWc: Platelet Distribution Width—coefficient of variation; MPV: Mean Platelet Volume.; 0, 7, 21, and 45 represent the blood sampling days.

## Data Availability

The data presented in this study are available on request from the corresponding authors due to privacy and confidentiality restrictions regarding the animal owners.
